# Single cell and immunity: Better understanding immune cell heterogeneities with single cell sequencing

**DOI:** 10.1002/ctm2.1159

**Published:** 2022-12-28

**Authors:** Dongsheng Chen, Yonglun Luo, Genhong Cheng

**Affiliations:** ^1^ Institute of Systems Medicine Chinese Academy of Medical Sciences & Peking Union Medical College Beijing China; ^2^ Suzhou Institute of Systems Medicine Suzhou China; ^3^ Lars Bolund Institute of Regenerative Medicine Qingdao‐Europe Advanced Institute for Life Sciences BGI‐Qingdao, BGI‐Shenzhen Qingdao China; ^4^ Department of Biomedicine Aarhus University Aarhus Denmark; ^5^ Department of Microbiology Immunology & Molecular Genetics University of California Los Angeles (UCLA), Los Angeles, California, USA

**Keywords:** cancer, immunity, infection, sequencing, single cell

## Abstract

Single‐cell sequencing has scientific impacts on better understanding the immunity. There is a rapid development in single cell‐based databases and analytic tools to provide the potential of clinical and translational discovery. The understanding of single‐cell based immunity needs a strong program and solid evidence of preclinical and clinical validation and evaluation. The current special topic issue on single cell and immunity aimed to provide a strong communication for the progress of single cell‐based studies on immune cell functional diversity in development and disease. The topic has a clear scope on the application of single cell sequencing to better understand immune cell heterogeneities, functions, cell–cell interactions, responses and regulatory roles in systems immunology and diseases.

The delicate balance of immunity is mediated by frequent crosstalk and complicated interactions between various cell types. Immune cells are highly heterogenous in cell types and activation status. Tissue and bulk cell sequencing methods capture the overall signatures from mixed samples, with the subtle gene signatures from rare yet functionally important cell types covered by abundant cell populations. Furthermore, intra‐ and inter‐cellular communications carrying essential clues for immune cell proliferation, differentiation, maturation, migration and transition are difficult to be characterised using traditional methods. The current special topic issue aimed to focus on the rapid progression of single cell‐based studies on infectious immunity, cancer immunology, innate immunity and immune system disorders, as shown in Figure 1.

**FIGURE 1:**
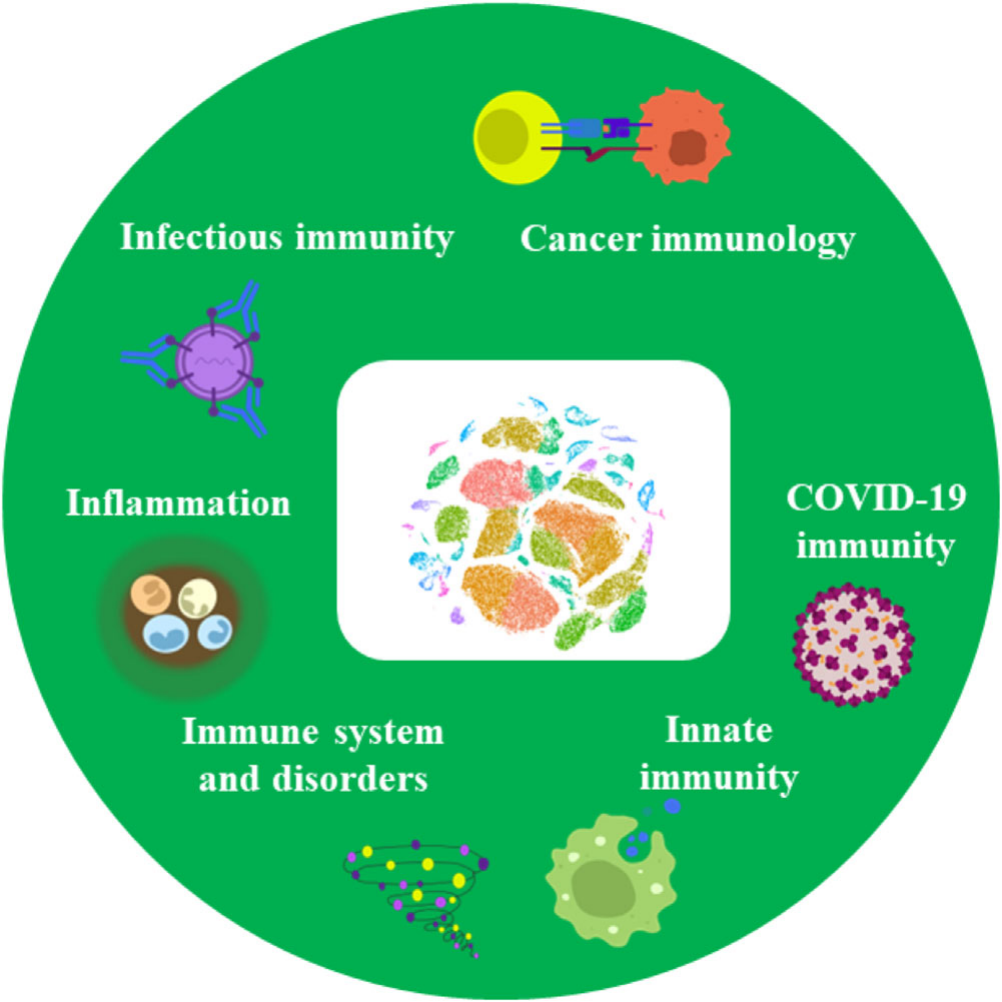
The topic on single cell and immunity is mainly focused on immune system disorders, infectious immunity, cancer immunity, inflammatory immunity, virus‐associated immunity, and innate immunity. Schematic diagrams were created with BioRender.com.

Single‐cell sequencing has scientific impacts on clinical and translational discovery. In the past decade, single‐cell RNA sequencing (scRNA‐seq) has triggered a true revolution in immunological research. As single‐cell molecular profiling tools, scRNA‐seq technologies provide a better understanding of the heterogeneity associated with individual immune cells and the responses at the molecular levels under physiological and pathological conditions.^1^ The single‐cell sequencing‐based studies have successfully identified new immune cell subsets and developmental trajectories.^2^ Infectious diseases and cancers pose urgent threat to human health. How to translate our understanding of molecular mechanism of immune regulation into clinical therapies remain a big challenge. With scRNA‐seq technology, major breakthroughs have been achieved. For examples, the distinct immune response pathways between infections with influenza A virus and severe acute respiratory syndrome coronavirus‐2 (SARS‐CoV‐2) were uncovered at single cell resolution.^3^ The SARS‐CoV‐2‐neutralizing antibodies were identified using scRNA‐seq of antigen‐enriched B cells of COVID‐19 patients.^4^ The cross‐tissue immune atlas and developmental immune atlas of humans were systematically constructed.^5^, ^6^ The scRNA‐seq technology has been proven to be a potent tool to reveal mechanisms of immune escape in tumour tissues.^7^

There is a rapid progress in single cell analytic tools. A wide variety of comprehensive online databases were developed for the storage and analysis of single cell data sets.^8^, ^9^, ^10^, ^11^, ^12^, ^13^ It is urgently needed to innovate more bioinformatics tools for data integration from a variety of technologies and data types. One crucial task is to standardize the annotation of immune cells and exploit the full spectrum of data types. This will enable the ability to collaborate on data analysis between academia, industry and clinical institutions. Thus, the coordinated effort from this special topic issue will advance our understanding of immunology research and therapies.

## CONFLICT OF INTEREST

The authors declare no conflict of interest.

## Data Availability

Data sharing is not applicable to this article as no new data were created or analysed in this study.
